# Raising awareness for rheumatic mitral valve disease

**DOI:** 10.21542/gcsp.2020.26

**Published:** 2020-11-30

**Authors:** Livia S.A. Passos, Maria Carmo P. Nunes, Peter Zilla, Magdi H. Yacoub, Elena Aikawa

**Affiliations:** 1 The Center for Excellence in Vascular Biology, Brigham and Women’s Hospital, Harvard Medical School, Boston, MA, USA; 2 Hospital das Clínicas e Faculdade de Medicina da Universidade Federal de Minas Gerais, Belo Horizonte, Minas Gerais, Brazil; 3 University of Cape Town, Cape Town, South Africa; 4 Imperial College London, London, UK

## Abstract

Rheumatic heart disease (RHD) is a major burden in low- to mid-income countries, where each year it accounts for over a million premature deaths associated with severe valve disease. Life-saving valve replacement procedures are not available to the majority of affected RHD patients, contributing to an increased risk of death in young adults and creating a devastating impact. In December 2017, a group of representatives of major cardiothoracic societies and industry, discussed the plight of the millions of patients who suffer from RHD. A comprehensive solution based on this global partnership was outlined in “The Cape Town Declaration on Access to Cardiac Surgery in the Developing World”. The key challenge in controlling RHD is related to identification and removal of barriers to the translation of existing knowledge into policy, programs, and practice to provide high-quality care for patients with RHD. This review provides an overview on RHD by emphasizing the disease medical and economic burdens worldwide, risk factors, recent advance for early disease detection, and overall preventive strategies.

## Introduction

Acute rheumatic fever (ARF) is the result of an abnormal response to pharyngitis caused by infection caused by a group A streptococcal infection in a genetically susceptible host^1–4^. The long-term damage to cardiac valves caused by ARF is known as rheumatic heart disease (RHD), which is an important cause of mortality in resource-poor settings around the world^4^.

Rheumatic heart disease (RHD) is a major burden in low- to mid-income countries, where it is the leading cause of cardiovascular death in children and young adults^5–7^. Severe valve disease, which is potentially amenable to intervention, remains strongly associated with mortality^6,8^, especially where life-saving cardiac surgery is either scarce or absent. Valve replacement surgeries are not available to the majority of affected RHD patients, contributing to an increased risk of death and major adverse outcomes^8–10^.

Although ARF has declined over the past 2 decades in industrialized countries, there are still a large number of chronic RHD cases, often complicated by chronic congestive heart failure and recurrent thromboembolic phenomena, both posing greater challenges for management^5,11^. Most deaths occur in young adults, who would otherwise be at the most productive years of their lives, indicating the devastating impact of this condition^9,12^. Additionally, RHD causes substantial disability in children and adults, which can affect quality of life and economic growth^12^.

Accordingly, in December 2017, on the occasion of 50^th^ anniversary of the world’s first heart transplant, a group of representatives including the major global and regional cardiothoracic societies, nongovernmental organizations and industry, discussed the plight of the millions of patients who suffer from RHD^10^. A comprehensive solution based on a global coalition aims to:

(1) advocate jointly-agreed prescription and audit policies to governments;

(2) engage industry for large-scale initiatives;

(3) incentivize donors to participate in programs, and

(4) recognize regional centers and training programs as outlined in “The Cape Town Declaration on Access to Cardiac Surgery in the Developing World”.

This strategy was a reaction to the slow progress in prevention in the past 15 years, implying that surgery will remain an integral part of the treatment of RHD for several generations. As such, it is imperative that this action is taken urgently.

The most important complication of RHD is valvular heart damage leading to altered hemodynamics, ventricular remodeling, and sequential heart failure^13,14^. Surgical interventions are required to replace or repair the damaged heart valves. In a small proportion of patients, balloon valvotomies could be an option and promising tailor-made transcatheter heart valve prostheses for rheumatic patients are in pre-clinical trials^15^. However, rates of the use of percutaneous and surgical interventions in resource-poor settings may be limited by the shortage of health facilities and trained staff^9,11,16^. Additionally, many patients with RHD are first diagnosed with advanced disease, at a stage when valve intervention there is limited impact on outcomes^9,12^.

Despite the observed progress in research on RHD pathogenesis, a number of key questions remain. Specifically, the underlying mechanisms involved in the development of severe valve dysfunction are not completely understood. There is significant variability in individual susceptibility to RHD, including higher frequency of disease in females. The cause of this association is not known, which warrants further investigation to understand sex specificity in the occurrence of RHD^7,17^. Furthermore, the burden of RHD in women is underappreciated, leading to high maternal and fetal mortality^9^.

The aim of this review is to provide an overview on RHD by raising awareness of the disease and its effects on medical and economic burdens worldwide, risk factors, recent advance for early disease detection, and overall preventive strategies.

### Epidemiology: challenges and global disease burden

There is increasing interest in RHD worldwide over the past decade, driven in part by the availability of echocardiography-based screening in countries in which the condition remains endemic^18,19^. Echocardiographic screening has played a pivotal role to estimate the burden of RHD and to guide policies for the control of disease^20–23^. The overall prevalence of subclinical RHD seen on echocardiography is seven to eight times higher than that of clinically manifested disease^24^. However, the majority of studies used echocardiographic screening that has been conducted in children attending schools, among which RHD may be less frequent than in the total population^23^. Indeed, emerging data suggest that RHD is detected more frequently in adults and children in community settings^25,26^.

The global burden of RHD is significant, especially in populations living in limited-resource countries^5^. A systematic literature review as part of the 2015 Global Burden of Disease study estimated about 33 million individuals (0.4% of the global population) are currently living with RHD. The number of disability-adjusted life-years due to RHD in 2015 was 10.5 million, accounting for 0.43% of global disability-adjusted life-years due to any other cause. Most disability-adjusted life-years due to RHD were the result of years of life lost, which indicated that premature death was a main cause of total health loss from RHD. The highest death rates were in the highest prevalence regions, and no significant decline in mortality over 1990 to 2015 was detected in a number of countries. The highest age-standardized mortality due to and prevalence of RHD was observed in Oceania, South Asia, and central sub-Saharan Africa^5^.

It is important to emphasize that much of the morbidity and mortality of RHD can be prevented by existing therapies^9^, but if left untreated, subsequent heart failure and death is almost inevitable. The REMEDY study (Global Rheumatic Heart Disease Registry) documented high rates of disability and premature death across African and Asian countries, which was largely attributable to advanced disease at the time of presentation^8^. This study confirmed that RHD is still a major cause of preventable death and suffering in children and young adults in low- and middle-income countries. Suboptimal utilization of secondary antibiotic prophylaxis, oral anticoagulation, and contraception, and variations in the use of percutaneous and surgical interventions by country income level contribute to high prevalence of major cardiovascular complications.

After a period of relative neglect, there has recently been a growing need to raise awareness of RHD and its devastating impact^6^. In 2015, a civil society movement, RHD Action, was launched to create awareness and support countries looking at addressing RHD^6^. More recently, the World Health Assembly adopted a resolution to reinvigorate global and national rheumatic fever and RHD prevention and control efforts.

The key challenge in controlling RHD is related to the identification and removal of barriers to the translation of existing knowledge into policy, programs, and practice^6^. There is good evidence that the successful elimination of RHD and relief from its debilitating consequences in those already affected can occur on the basis of comprehensive programs^5^. The goal of such programs must be to improve the delivery of proven control strategies that were shown to affect people and communities.

### Demographical and environmental risk factors

ARF largely occurs in children, particularly those living in socioeconomic deprivation with highest incidence among children aged 10–14 years, followed by those aged 5–9 years^27^. Recurrent episodes often affect slightly older children, adolescents and young adults but are rarely observed beyond the age of 35–40 years. In contrast, peak prevalence of RHD is in adulthood, usually between the ages of 25 years and 45 years^4^. In endemic areas, the disease may progress more rapidly with children presenting with severe valvular lesions, whereas in developed countries, the progression of disease is more indolent and manifests at older ages^28^.

ARF is equally common in males and females, whereas RHD occurs more commonly in females. In the published literature of the current era, clinically detected RHD is most commonly diagnosed in individuals aged 20 to 50 years with nearly two-thirds of cases occurring in females^28^. Women are not only prone to have a higher incidence of rheumatic mitral stenosis but are also at an increased risk of death in the setting of RHD^29^. The particular burden of RHD on women needs more attention. RHD in pregnancy is becoming increasingly recognized. In the REMEDY study, 60% of patients were women, most of childbearing age, and only 4% were on contraceptives. Pregnancy in women with RHD is a hidden cause of maternal as well as fetal mortality^9,12^. Data from endemic countries suggest that RHD is a leading cause of indirect obstetric death, which in turn accounts for 25% of all maternal deaths in developing countries^30^. This effect relates to the worsening of pre-existing disease as a result of hemodynamic changes that occur during pregnancy, rather than any increase in susceptibility to ARF or RHD because of pregnancy.

Overall, RHD is a disease of poverty, overcrowding, poor sanitation, and inadequate access to health care^31^. Several studies have confirmed the significant association of low socioeconomic status and increased prevalence of RHD (Table 1), indicating that socioeconomic factors play an important role in pathophysiology of RHD^32^.

**Table 1:** Association between socioeconomic factors and the prevalence of RHD in different populations.

**Reference(Author/year)**	**Country**	**N**o **of individuals**	**Socio-economic characteristics**
Chun, 1984^49^	USA,Hawaii^*^	104	Low socio-economic status
Vlajinac, 1991^50^	Yugoslavia	148	Home dampness, Low education of mother^**^
Steer, 1999^51^	Samoa	Survey of RHD in 8767 school children	Rural background
Dobson, 2012^52^	Fiji	80	Maternal unemployment
Riaz, 2013^53^	Bangladesh	103	Illiteracy and overcrowding
Beg, 2016^54^	Pakistan	130	Low economic status, poor hygienic conditions, and illiteracy
Shrestha, 2016^55^	Nepal	53 subclinical RHD	Governmental schools and unemployment of parents
Gurney, 2016^27^	New Zealand	711 with ARF	Deprivation strata

**Notes.**

* Among ethnic groups of Hawaii.

** Less than 4 years of elementary school.

The vast majority of differences in risk between populations around the world can be explained by environmental factors. The relative contribution of each of these individual risks is difficult to elucidate given that many of them overlap and most are associated with poverty and economic disadvantage. Household overcrowding is perhaps the most described risk factor among all of the environmental factors, which is amenable to improvement^4^.

### Presentation and diagnostic of RHD

Clinical myocarditis during initial ARF is a pan-myocarditis with resolution most often in the first year following acute stage^2^. In contrast, chronic RHD is almost exclusively dominated by valvular pathology. Although all four valves can be affected by rheumatic fever, left-sided valvular involvement is most commonly seen. The mitral valve is affected in almost all RHD cases^13^, with regurgitation in the early stages, and stenosis in later stages. The aortic valve is involved in 20–30% of cases^13^, and the tricuspid valve can also be affected, albeit less commonly. The interval between the initial episode of rheumatic fever and clinical evidence of valve disease is variable, ranging from a few years to more than 20 years. However, RHD has a more aggressive course in sub-Saharan Africa and other low-income regions^33^.

Both ARF and RHD result in characteristic morphological changes of the mitral valve. The morphological characteristics of ARF are extreme annular dilatation (typically from 23 mm to 37 mm) and prolapse of the anterior leaflet due to elongated but hardly ever ruptured chordae. Together with a largely normal posterior leaflet with only minimal chordal shortening due to the annular dilatation, the echocardiographic hallmark of ARF is a posteriorily-directed regurgitation jet. Typically, imaging diagnostic features are thickening at the leaflet edges and subvalvular apparatus, shortened chordae tendineae, commissural fusion, calcification, and restricted leaflet motion. Usually, the base and mid-sections of the leaflets move toward the ventricular apex, while the motion of the leaflet tips is restricted because of fusion of the anterior and posterior leaflets along the medial and lateral commissures^34^.

Echocardiography is the primary tool for evaluation patients with RHD^35^. Numerous studies over the last 2 decades have addressed the role of echocardiography in the diagnosis of ARF and chronic RHD^2^. Screening echocardiography to identify RHD has emerged as a powerful tool for active case finding, epidemiology, and advocacy^20–23, 36–39^. There is increasing evidence demonstrating that echocardiography-based screening may be the most promising approach for preventing disease progression^2,40^. However, the natural history of RHD in children with subclinical abnormalities detected by echocardiography remains unknown^41,42^. The challenge is how to manage clinically children with silent RHD, whose prevalence is substantially higher than the cases of overt RHD^24^.

**Figure 1:**
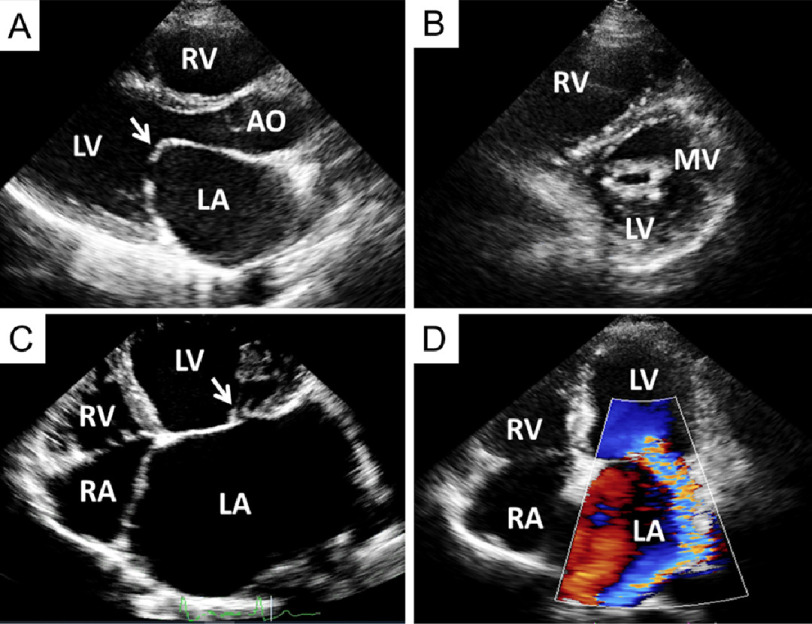
Transthoracic echocardiography from a patient with severe mitral stenosis in parasternal long-axis (A) and short-axis (B) views. Both leaflets are thickened with a pliable anterior leaflet on the long-axis view and fusion of both commissures on the short-axis view. The anterior leaflet has been described as opening in a “hockey stick” appearance in parasternal long axis view (arrow). Transthoracic echocardiography from a patient with severe mitral regurgitation, apical four-chamber view. A coaptation defect of mitral valve can be detected (arrow) with severely dilated left atrium (C). Color flow mapping showing severe mitral regurgitation (D). LA = left atrium; LV = left ventricle; Ao = aorta; RV = right ventricle MV = mitral valve area, RA = right atrium.

Echocardiography has become a reliable method for assessment of valve lesions secondary to RHD, which provides the anatomic changes, and accurately quantifies the severity of the valve stenosis or regurgitation^35,43^. In rheumatic mitral stenosis, echocardiography allows for detailed evaluation of mitral valve morphology, including assessment of leaflet thickness, leaflet mobility, the degree of calcification, and the extent of subvalvular involvement (Figure 1A–D). Percutaneous mitral valvuloplasty is the treatment of choice for patients with mitral stenosis, and echocardiography plays a crucial role in the assessment of severity of mitral stenosis and suitability for the procedure^44,45^.

Mitral valve regurgitation is the most common abnormality seen in patients with RHD^18,33,46^, particularly in the early stages. Mitral regurgitation (MR) primarily results from morphological changes that reflect chronic scarring of the mitral valve leaflets and mitral valve apparatus. The typical echocardiographic feature of MR is valvular thickening, particularly at the free edge of the leaflets. Chordal thickening is also a common characteristic of rheumatic involvement and results in the restricted movement of the mitral leaflets. The coaptation defect is typically associated with significant rheumatic MR (Figure 1B–D). Chordal rupture resulting in flail leaflet with severe MR may be seen in ARF^33^.

**Figure 2:**
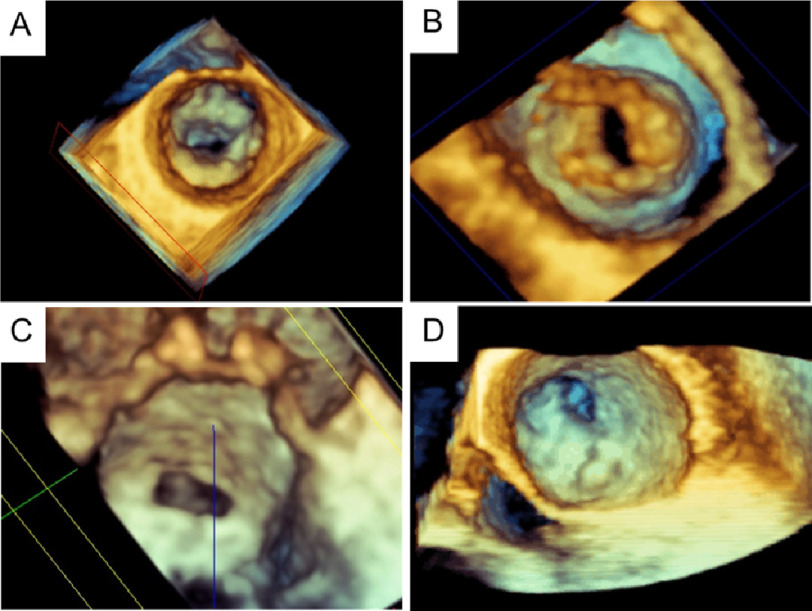
Three-dimensional transesophageal echocardiography images of rheumatic mitral stenosis. Mitral valve seen from the left atrium (A). En face view of the mitral valve from the left ventricle (B). Rheumatic mitral stenosis with symmetric commissural thickening. Severe rheumatic mitral stenosis with significant thickening of anterolateral commissure, leading to an eccentric valve orifice (D).

Three-dimensional echocardiography imaging is increasingly being used for evaluating mitral valve morphology, specifically the pattern of rheumatic commissural involvement, as well as for determining the time and the appropriate therapy for valve intervention (Figure 2A–D).

### Potential strategies for RHD control

Prevention strategies are the most appealing option for sustainable disease control in developing nations. The WHO global action plan is to reduce the premature mortality of non-communicable in 25% by the year of 2025, and the control and prevention of RHD has great impact in achieving this goal^47^. The preventive strategies include primordial, primary, secondary, and tertiary prevention, each of which is specific for certain susceptible groups and situations.

Primordial prevention applies to the general population, especially in socioeconomically disadvantaged contexts, and involves prophylactic strategies to avoid streptococcal infection. This prevention was the responsible for the decline in cases of acute rheumatic fever and RHD in most of the countries in the 20th even before the introduction of antibiotics^48^. The precariousness of this type of prevention in resource-poor settings justifies the fact that rheumatic fever is still a major public health challenge among these populations^4^. Improving community awareness has been demonstrated by programs incorporating public education campaigns into their RHD control strategies as a vital element.

Sore throat detection and treatment and prevention of recurrent streptococcal infection with long-term antibiotics are other approaches to reduce ARF and RHD burden^3^. However, it is now well known that there is poor use of established preventive and control strategies in populations with RHD. The barriers to uptake of simple, inexpensive treatments such as penicillin are numerous but mainly reflect the lack of systems to identify and to track patients with RHD in the community^4^. Establishment of ARF and RHD registers for this purpose has been advocated as an important step towards disease control. Case-finding, referral and treatment monitoring as part of school health or other child health programs is possible with education of providers and additional funding.

**Figure 3:**
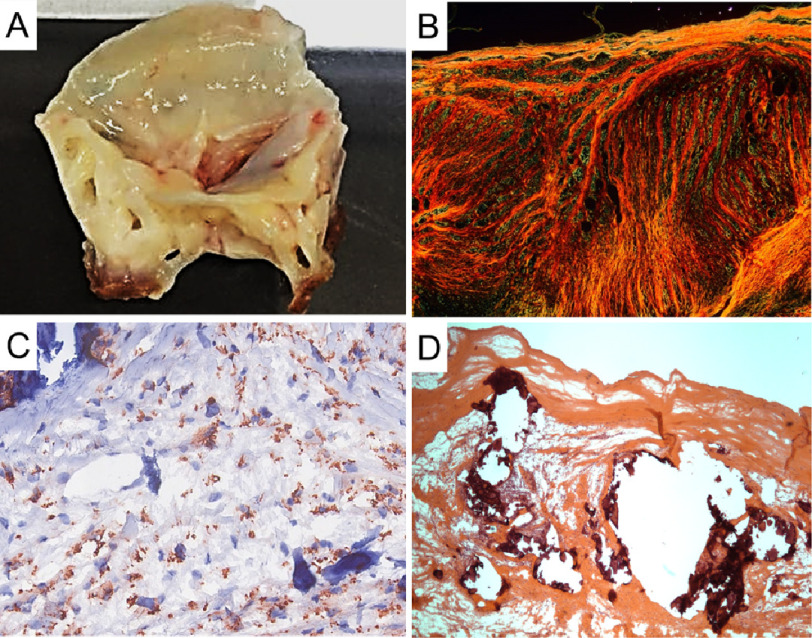
Gross and histological pathomorphological aspects of a rheumatic mitral valve in an end state of the disease. (A) Mitral valves are thick and stiff and show scar retraction. (B) Representative image of Picrosirius red staining showing intense collagen deposition e high degree of fibrosis. (C) Representative immunohistochemistry image for CD68+ cells evidencing a high frequency of macrophages in the inflammatory infiltrate. (D) Representative image of Hematoxylin and Eosin staining of anterior mitral valve leaflets showing presence of nodular calcification (deep purple) and intense inflammatory infiltrate.

### Final considerations

RHD has largely disappeared from wealthy countries, which may be a consequence of improvements in socioeconomic conditions and the widespread use of penicillin G benzathine to treat streptococcal pharyngitis. On the other hand, RHD is the major cause of cardiovascular death in children and young adults in developing countries and its treatment remains a challenge.

Although echocardiographic screening has played a major role to early diagnosis of RHD, key questions have been raised on optimal therapy for patients already suffering from the disease. The lack of access to cardiac surgery as a life-saving treatment continues to represent a fatal endpoint of the disease for millions of patients. Most affected patients present with severe valve disease, when timely valve intervention may improve outcomes in these patients.

Strategies to provide high-quality tertiary care for patients with RHD should be proposed, including sustainable cardiac surgical services. An integrated approach that supports training new generation of surgeons at established cardiac surgical centers in high-income countries would oversee the development of cardiac surgery in the low- to mid-income countries. Efforts should be accelerated towards tailor-made engineering solutions, including tissue engineered and transcatheter valves to treat young patients with RHD.

Renewed interest in RHD and a first global effort to unite the scientific potential of high-income regions with the clinical experience of low-and mid-income countries promise to breathe new life into the combat of an old health problem. In industrialized countries the socioeconomic developments of the 1950s and 1960s led to the near abolition of RHD, which had continued unabated in overcrowding and poverty. Yet, in the absence of cardiac surgical capabilities even the extent to which RHD requires surgical intervention had remained in the dark. This changed with the process initiated by the Cape Town Declaration^10^. Beyond creating a global structure that promises to unite the research capacity of developed countries with the clinical exposure of developing countries, one of the significant secondary gains of the Cape Town Process was to elucidate the true magnitude of the clinical problem.

For the first time, a multi-nation effort representing almost 4 billion people of 16 countries showed the annual need for life-saving heart valve surgery for RHD. As the umbrella structure created under the Cape Town Declaration is based on 5 pillars of which two are sites with a high clinical burden of RHD and one is a renowned academic institution in a high-income country, research efforts can be expected to see a distinct boost.

Together with the emerging medical science and newly awakened global policies to tackle RHD, chances have never been so great to end a health debacle that has targeted the poorest and weakest of societies for too long.

## Funding

Dr. Elena Aikawa is supported by National Institutes of Health (NIH) grants R01HL 136431, R01HL 141917 and R01HL 147095.

## Disclosures

None.
